# Alterations in gut microbiota and metabolite profiles in patients with infantile cholestasis

**DOI:** 10.1186/s12866-023-03115-1

**Published:** 2023-11-18

**Authors:** Meng Jin, Jinghua Cui, Huijuan Ning, Meijuan Wang, Wenwen Liu, Kunyu Yao, Jing Yuan, Xuemei Zhong

**Affiliations:** 1 Gastroenterology Department, Children’s Hospital Capital Institute of Pediatrics, Beijing, 100020 China; 2 Bacteriology Department, Capital Institute of Pediatrics, Beijing, 100020 China

**Keywords:** Infantile cholestasis (IC), Gut microbiota, Microbiota-derived metabolites, *Ruminococcus*, *Butyrivibrio*, *Veillonella*

## Abstract

**Background:**

Infantile cholestasis (IC) is the most common hepatobiliary disease in infants, resulting in elevated direct bilirubin levels. Indeed, hepatointestinal circulation impacts bile acid and bilirubin metabolism. This study evaluates changes in the gut microbiota composition in children with IC and identifies abnormal metabolite profiles associated with microbial alterations.

**Results:**

The gut microbiota in the IC group exhibits the higher abundance of *Veillonella*, *Streptococcus* and *Clostridium spp*. (*P* < 0.05), compared to healthy infants (CON) group. Moreover, the abundance of *Ruminococcus*, *Vibrio butyricum*, *Eubacterium coprostanogenes group*, *Intestinibacter*, and *Faecalibacterium* were lower (*P* < 0.05). In terms of microbiota-derived metabolites, the levels of fatty acids (palmitoleic, α-linolenic, arachidonic, and linoleic) (*P* < 0.05) increased and the levels of amino acids decreased in IC group. Furthermore, the abundances of *Ruminococcus*, *Eubacterium coprostanoligenes group*, *Intestinibacter* and *Butyrivibrio* are positively correlated with proline, asparagine and aspartic acid, but negatively correlated with the α-linolenic acid, linoleic acid, palmitoleic acid and arachidonic acid. For analysis of the relationship between the microbiota and clinical index, it was found that the abundance of *Veillonella* and *Streptococcus* was positively correlated with serum bile acid content (*P* < 0.05), while APTT, PT and INR were negatively correlated with *Faecalibalum* and *Ruminococcus* (*P* < 0.05).

**Conclusion:**

Microbiota dysbiosis happened in IC children, which also can lead to the abnormal metabolism, thus obstructing the absorption of enteral nutrition and aggravating liver cell damage. *Veillonella, Ruminococcus* and *Butyrivibrio* may be important microbiome related with IC and need further research.

**Supplementary Information:**

The online version contains supplementary material available at 10.1186/s12866-023-03115-1.

## Background

Infantile cholestasis refers to abnormal bile formation, secretion, and excretion into bile canaliculi, resulting in increased blood direct bilirubin levels, is the most common hepatobiliary disease in infants, with an incidence rate of approximately 1/2,500 [1]. The common causes include biliary atresia (25–40%), monogenic cholestasis (25%), and other unknown or multifactorial factors [2, 3]. The diagnostic criteria for cholestasis defined by the North American Society for Pediatric Gastroenterology, Hepatology and Nutrition (NASPGHAN) guideline issued in 2004 [4] are: total bilirubin concentration (TBil) < 85.5 μmol/L, direct bilirubin concentration (DBil) > 17.1 μmol/L or TBil ≥ 85.5 μmol/L, and DBil:TBil ratio > 20%. Meanwhile, the most recent Guideline for the Evaluation of Cholestatic Jaundice in Infants jointly published by NASPGHAN and the European Society for Pediatric Gastroenterology Hepatology and Nutrition (ESPGHAN) in 2017, indicated that infants with a serum DBil > 17.1 mmol/L should be diagnosed with cholestasis regardless of whether TBil is increased.

Currently, IC is the leading cause of hospitalizations among children with liver diseases. Accordingly, considerable attention has been devoted to investigating IC, with a primary focus on etiology and differential diagnosis. Moreover, due to the development of genetic diagnosis methods, approximately one third of infants with intrahepatic cholestasis have received definitive diagnoses via gene detection, facilitating the administration of precise treatment regimens [5]. In addition, several studies have demonstrated a relationship between hepatointestinal circulation and bile acid and bilirubin metabolism [6–8]. Hence, research focused on identifying specific intestinal biomarkers has attracted increasing attention and is expected to improve early diagnosis and treatment. The liver and the gastrointestinal (GI) tract have a shared embryonic origin with certain ‘intrinsic’ anatomical and physiological connections [6]. Intestinal metabolites (including intestinal toxins and microbiota-derived metabolites) that enter the liver through the superior and inferior mesenteric veins, as well as the portal vein during gut microbiota dysbiosis can alter liver function by inducing hepatocyte inflammatory injury and activating hepatic immune responses. The lack of bile acid within the intestinal lumen serves as the primary mechanism by which cholestasis causes gut microbiota dysbiosis. This disrupts the mechanical, chemical, immunological, and biological barriers of the intestinal mucosa, facilitating the entry of bacterial metabolites and endotoxins into the bloodstream, resulting in organ and tissue injury [7–10]. As such, the bidirectional communication between the gut microbiota and the liver via gut microbiota-derived metabolites is expected to become a new target for the diagnosis, treatment, and prevention of liver diseases due to its important roles in various liver diseases, such as fatty liver disease and cholestatic hepatopathy [11].

The current study analyzed changes in the composition and abundance of gut microbiota in healthy infants and patients with IC via high-throughput sequencing (HTS) of 16S rRNA. In addition, we performed non-targeted metabolomics analysis on patients with IC of unknown etiology via gas chromatography-time-of-flight mass spectrometry (GC-TOF/MS) to obtain abnormal gut microbiota-derived metabolite profiles. Our study aims to identify specific microbiota or metabolite markers associated with the onset of IC by comparing changes in the gut microbiota composition and abnormal metabolites between IC patients and healthy infants. We also aim to elucidate the main metabolic pathways in gut microbiota during the development of IC to provide insights regarding IC pathogenesis that will inform the development of new diagnostic and therapeutic strategies based on host–microbe interactions.

## Results

### Description of patient characteristics and clinical indicators

We recruited a total of 20 pediatric patients with IC (11 males and 9 females; average age: 2.98 months; average duration of disease: 1.4 months) admitted to our hospital from January 2020 to June 2021 who fulfilled the following criteria: aged < 1 year, no antibiotics or probiotics administered over the preceding 2 weeks, full-term infants born by vaginal delivery, exclusively breastfed before disease onset, and met the diagnostic criteria for cholestasis at admission. Table 1 summarizes the analysis of clinical indicators, of which alanine amino transferase (ALT), aspartate transferase (AST), and glutamyl transpeptidase (GGT) are enzymatic indicators of hepatocellular injury. In the IC group, two patients had normal ALT concentrations, one had normal AST concentrations, four had normal GGT concentrations, and one had normal ALT, AST, and GGT concentrations; the remaining pediatric patients exhibited varying degrees of increased transaminase concentrations, implying that most patients in this group had hepatocellular injury. All IC patients had substantially higher than normal TBIL, DBIL, and TBA values, which represent the severity of intrahepatic or extrahepatic cholestasis. Patients with severe hepatocellular injury have a higher risk of hemorrhage. However, the prothrombin time (PT), activated partial thromboplastin time (APTT), and International normalized ratio (INR) of patients with IC fell within normal ranges. Meanwhile, three patients with IC exhibited a mild elevation of the blood ammonia concentration with no apparent abnormality. An elevated lactate concentration often suggests the possibility of inherited metabolic liver disease; however, patients with IC in this study did not show a significant increase in lactate concentration. The known causes of IC in the patients include biliary atresia (one case), defects in *MYO5B* (one case), and citrin deficiency (two cases), cytomegalovirus infection (two case), respiratory tract infection and urinary tract infection (six cases), while the etiology for the remaining 8 patients was unknown (Table 1). Fecal specimens from 20 age-matched, healthy infants at the Department of Health of our hospital were also collected. The 40 specimens were collected and divided into the IC and healthy control (CON) groups, respectively.

**Table 1:** Demographic and clinical characteristics of pediatric patients in the infantile cholestasis group

NO	Sex	Age (m)	Course of disease (m)		ALT U/l (10–44)	AST U/l (10–50)	ALB g/l (35–50)
1	F	1.96	1.6		162.1	79.3	44.4
2	F	2.5	2		420	475.6	40.5
3	M	2.43	2		31.8	69	30.3
4	M	1.73	1		88.7	115.3	41.2
5	F	2	1		294.6	335.6	39.1
6	M	5.96	1		178.8	178.4	46.8
7	M	2.63	2		78.2	71.1	38.5
8	M	1.66	1.2		175.9	250.5	38.6
9	F	1.33	1		51.4	102.6	34.6
10	F	2	1		23	44	73
11	M	2	1		93.8	191.1	37.2
12	F	6.13	1		229.6	332.6	37.1
13	F	3	2.5		135	273.7	40.5
14	M	2.83	1		40.7	79.4	37.3
15	F	3	2		56	83	40
16	M	1	1		192	250	38.1
17	F	11	1		192	250	45.1
18	M	2.46	1		103.7	138.6	38.2
19	M	1.86	1		153.1	107.8	44.8
20	M	2.1	2		369	377.8	41.1
	M/F 11:9	Average: 2.98	Average: 1.4		147.38 ± 108.1	179.12 ± 123.3	41.32 ± 8.1
NO	GGT U/l (9–50)	TBIL μmol/l (3.4–20.0)	DBIL μmol/l (< 3.4)		TBA μmol/l (0–10)	WBC 10^9^/l (4.3–14.2)	Hb g/l (110–150)
1	155	103	74.4		99.1	9.61	116
2	82	213.6	123.8		157	11.44	90
3	170	104.2	62.8		62.2	11.25	80
4	142	173.6	107		107.4	7.02	128
5	272	68.7	37.7		48.6	8.73	79
6	2547	317.5	183.3		246.1	19.95	106
7	118	72.39	44.78		74.4	9.2	100
8	119	243.7	139.5		101.5	20.71	102
9	234	221.2	99.6		112.4	9.54	126
10	13	166.3	94.8		347.1	6.31	125
11	192	118.7	74.1		95.9	12.11	99
12	36	162.8	83.9		431.2	19.29	65
13	283	147.2	85.2		122.5	17.36	122
14	231	111.4	57.2		123.6	8.44	112
15	123	58.7	43.8		57.7	6.15	108
16	27	61.2	34.6		58.6	12.22	90
17	27	56.1	34.6		352.8	14.72	104
18	766	168.2	99		179.6	9.71	102
19	43	188.8	86.5		94.6	12.55	107
20	59	243.7	138.6		75.9	11.33	108
	287.4 ± 543.2	142.74 ± 81.6	80.04 ± 46.04		140.96 ± 113.44	11.88 ± 4.3	103.45 ± 16.18
NO	PLT 10^9^/l (183–614)	CRP mg/l (< 8)	PT s (10–13)	APTT s (22.3–32.5)	INR (0.9–1.2)	BA μmmol/l (11–40)	Lac mmol/l (0.5–2.2)
1	326	0.5	11.4	29.6	0.99	98.21	2.7
2	596	0.42	10.5	34.2	0.91	10	1.4
3	526	0.39	14.2	48.9	1.26	71.93	1.9
4	288	0.47	13.3	54.6	1.17	10	1.8
5	229	3.6	9.9	31.2	0.85	47.92	2.8
6	330	0.69	10.3	27.6	0.89	70.16	1.6
7	480	0.43	10.9	31.3	0.94	23.2	2.9
8	448	0.5	10.9	36.1	0.94	7.48	2
9	338	3	11.2	36.9	0.97	46.47	2.2
10	410	0.78	10.5	24.5	0.91	38.19	1.7
11	570	0.43	11.5	33.2	1	17.72	0.6
12	381	0.5	11	24.8	0.95	23.8	2.1
13	396	1	11.7	34.9	1.02	54.04	3.3
14	350	0.44	12.8	38.7	1.12	46.8	1.6
15	355	0.43	11.5	29.3	1	46.44	0.9
16	383	0.5	10.3	27.5	0.9	49.5	1.6
17	337	0.43	10.2	26.4	0.88	51.89	1.6
18	457	0.5	9.2	27.1	0.78	29.3	1.7
19	449	0.45	10.5	26.4	0.91	54.64	1.8
20	533	0.78	11.9	31.5	1.04	56	3.1
	409.1 ± 94.56	0.81 ± 0.85	11.19 ± 1.1	32.74 ± 7.53	0.97 ± 0.11	42.68 ± 22.67	1.97 ± 0.68

Continuous variables are expressed as mean ± standard deviation

*Abbreviations NO* number, *M* Male, *F* Female, *m* month, *ALT* Alanine aminotransferase, *AST* Aspartate aminotransferase *ALB* Albumin, *GGT* Glutamyl transpeptidase *TBIL*: Total bilirubin DBIL Direct bilirubin, *TBA* Total bile acid, *WBC* white blood cell count, *Hb* Hemoglobin, *PLT* Platelet count, *CRP* C-reactive protein, *PT* Prothrombin time, *APTT* Activated partial thromboplastin time, *INR* International normalized ratio, *BA* Blood ammonia, *Lac* Blood lactic acid

Reference values are in parentheses

### Characterization of gut microbiota in infants with IC

**Fig. 1:**
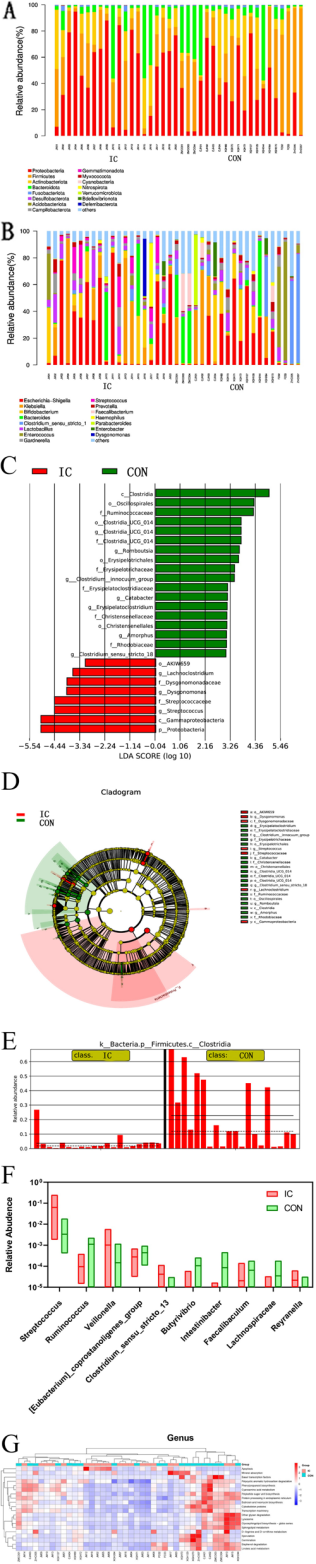
Characteristics of gut microbiota in infantile cholestasis (IC; *n* = 20) and control (CON; *n* = 20) groups. **A**. Distribution of gut microbiota at the phylum level in 40 specimens; **B**. distribution of gut microbiota at the genus level in 40 samples; **C**. score plot of differential species showing linear discriminant analysis of differentially abundant genera between the two groups; **D**. annotated cladogram of differential species showing statistically significant taxa between the two groups; 1E. relative abundance histogram. Solid line represents the mean relative abundance, dashed line represents the median relative abundance; **F**. distribution of gut microbiota with significant differences in relative abundance between the two groups; **G**. Heatmap of KEGG pathway enrichment analysis

The bioinformatics analysis of 16S rRNA reads from fecal specimens from the 40 patients detected 33 phyla, 77 classes, 173 orders, 286 families, and 554 genera. Figure 1A shows the top 15 most abundant bacterial phyla, mainly including *Proteobacteria*, *Firmicutes*, *Actinobacteriota*, *Bacteroidota*, and *Fusobacteriota* (Fig. 1A). At the genus level, it mainly includes 15 high abundance bacterial genera, such as *Escherichia-Shigella*, *Klebsiella*, *Bifidobacterium*, *Bacteroides*, *Clostridium-*sensu*-stricto* and so on (Fig. 1B). The OTU clustering identified 2,917 genera in the IC group and 2,966 genera in the CON group, with 2,255 shared between the groups. The Shannon and Chao indices of alpha diversity index between the IC and CON groups showed no statistically significant effect (Supplementary Fig. 1A-B), and the beta diversity showed no differences in microbial diversity (Supplementary Fig. 1C). LEfSe analysis reslut (Fig. 1C-E) shows that the abundance of *Proteobacteria,* *Gammaproteobacteria*, *Streptococcus,* and *Dysgonomonas* was relatively high in the IC group, while that of *Clostridia*, *Oscillospirales,* and *Ruminococcaceae* was relatively high in the CON group. Additionally, different microbiota was observed at the genus level between the two groups. That is, in top 10, the gut microbiota of IC patients showed that the abundance of *Streptococcus*, *Veillonell*a and *Clostridium stenosum* was significantly higher than that of CON group (*P* < 0.05). However, the abundance of *Ruminococcus*, *Vibrio butyricus*, *Colidextribacter*, *Eubacterium coprostanoligenes group*, *Intestinibacter*, *Faecalibaculum*, and *Spirillum* decreased (*P* < 0.05) (Fig. 1F). The heatmap of KEGG pathway enrichment analysis through these microbiota showed that the enriched pathways based on the 16S rRNA sequences of fecal specimens from each group could not be clustered (Fig. 1G). However, the microbiota of the patients with IC (7/20) whose ALT and AST concentrations twice that of the upper limit of the normal ranges, had high expression of apoptosis-associated pathway.

### Characterization of metabolites from stool of patients with IC of unknown etiology

**Fig. 2:**
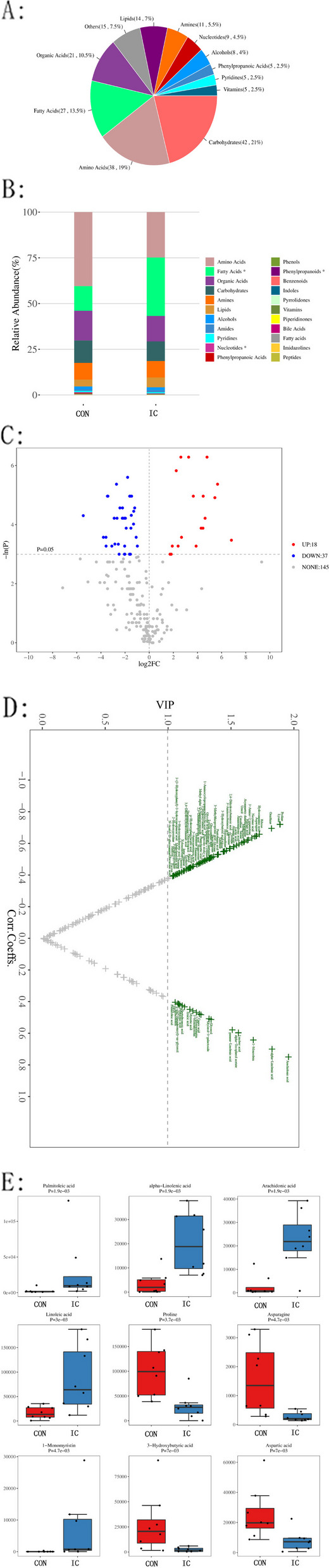
Profiling of gut microbiota-derived metabolites in the infantile cholestasis (IC) and control (CON) groups. **A** Categorical and quantitative distribution of metabolites across all samples; **B**. Distribution of metabolites in different groups; **C**. univariate statistical analysis of differential metabolites. Red dots on the right: upregulated metabolites in the IC compared to CON group, blue dots on the left: downregulated metabolites, gray dots (NONE): metabolites that did not meet the threshold requirements; **D**. multivariate statistical analysis of differential metabolites; *x*-axis: Pearson's correlation coefficients between each metabolite and first principal component (P1), *y*-axis: contribution of metabolites to the model discrimination (variable importance in projection; VIP). Green dots: metabolites with VIP score > 1; 2E. box plot showing the top nine differential metabolites

Sixteen samples (eight randomly selected samples from healthy infants and eight patients with IC of unknown etiology after genetic testing) were submitted for metabolic profiling. Two-hundred metabolites were identified that were further classified into 12 categories, including carbohydrates (42, 21%), amino acids (38, 19%), fatty acids (27, 13.5%), organic acids (21, 10.5%) and so on (Fig. 2A). Significant differences were detected in gut microbiota-derived metabolites, including fatty acids, phenylpropanoids, and nucleotides (P < 0.05) (Fig. 2B).

In the univariate analysis, we adopted the following threshold values in the volcano plot: 1) *P* < 0.05; 2) Absolute value of |log_2_FC|> 1 (FC: fold change between groups). In the univariate volcano plot, the highlighted dots in the upper-right and upper-left corners represent upregulated and downregulated metabolites, respectively, compared to the CON group. The univariate analysis identified 55 differential metabolites that met the selection criteria (Fig. 2C). The multivariate analysis based on the contribution (variable importance in projection, VIP) to group separation in the orthogonal projections to latent structures discriminant analysis (OPLS-DA) model and reliability (correlation coefficients) of metabolites identified 86 differential metabolites (Fig. 2D). A total of 46 reliable and biologically significant differential metabolites were selected. However, our analysis of the top nine most significant differential metabolites revealed that the IC group had higher intestinal concentrations of fatty acids (including palmitoleic, alpha-linolenic, arachidonic, and linoleic acids) and lipids (1-monomyristin), as well as lower concentrations of certain amino acids (including asparagine, aspartic acid, and proline) and organic acids (3-hydroxybutyric acid) than the CON group (Fig. 2E).

### Metabolic pathway enrichment analysis of gut microbiota in patients with IC

**Fig. 3:**
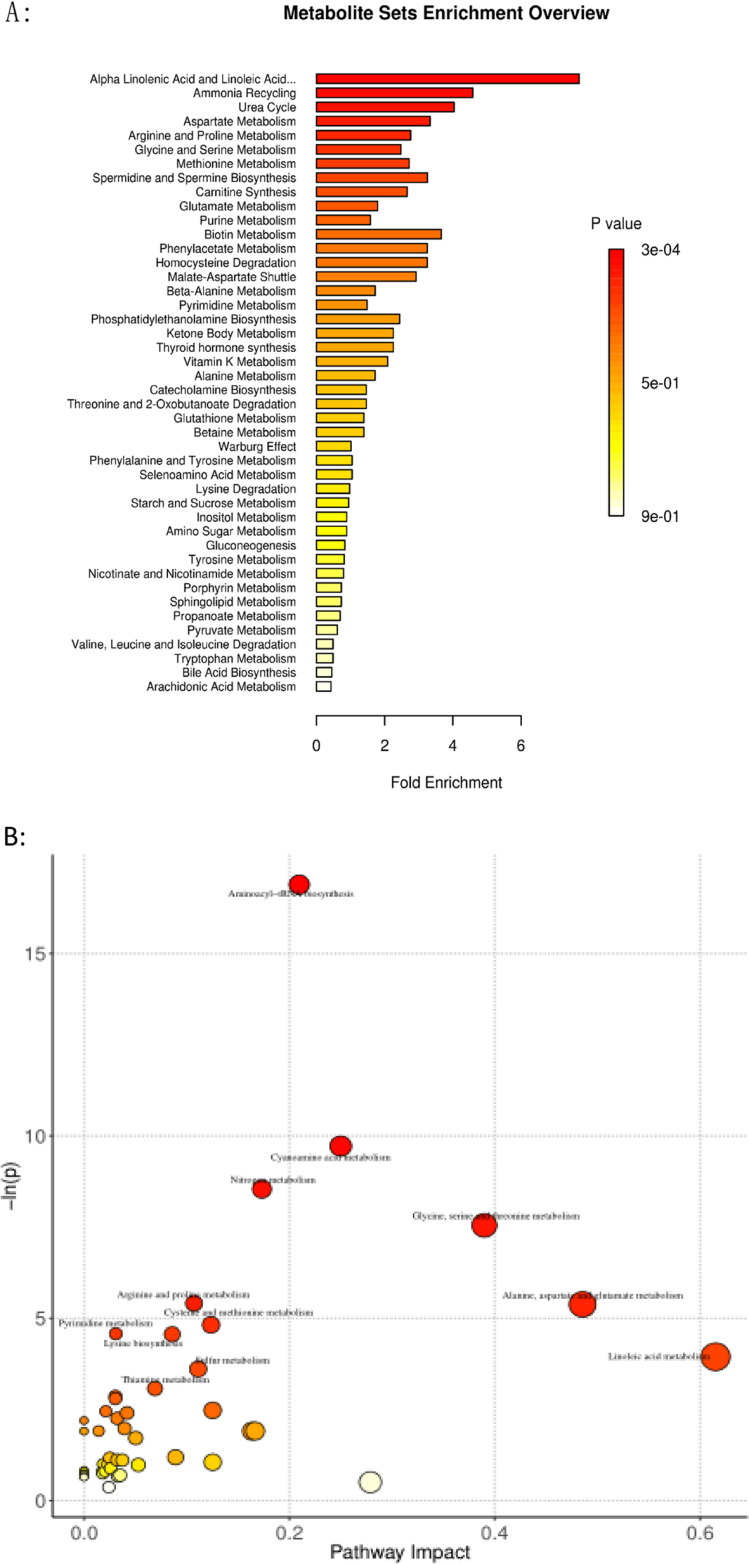
Metabolic pathway enrichment analysis of fecal samples in healthy infants and infants with infantile cholestasis(IC). **A** Pathway enrichment analysis for predicted metabolite sets; colors and length of horizontal bars represent the *P*-values of pathway enrichment and fold enrichment, respectively; 3. bubble map of metabolic pathway enrichment analysis

The pathway enrichment analysis using the pathway-associated metabolite sets (Small Molecule Pathway Database, SMPDB) (Fig. 3A) showed that the differential metabolites were primarily enriched in α-linolenic and linoleic acid metabolism, ammonia recycling, urea cycle, and aspartate, arginine, and proline metabolism. Figure 3B suggests that linoleic acid metabolism has the greatest impact on all metabolic pathways.

### Correlation analysis of gut microbiota with metabolites from stools, and clinical indicators

**Fig. 4:**
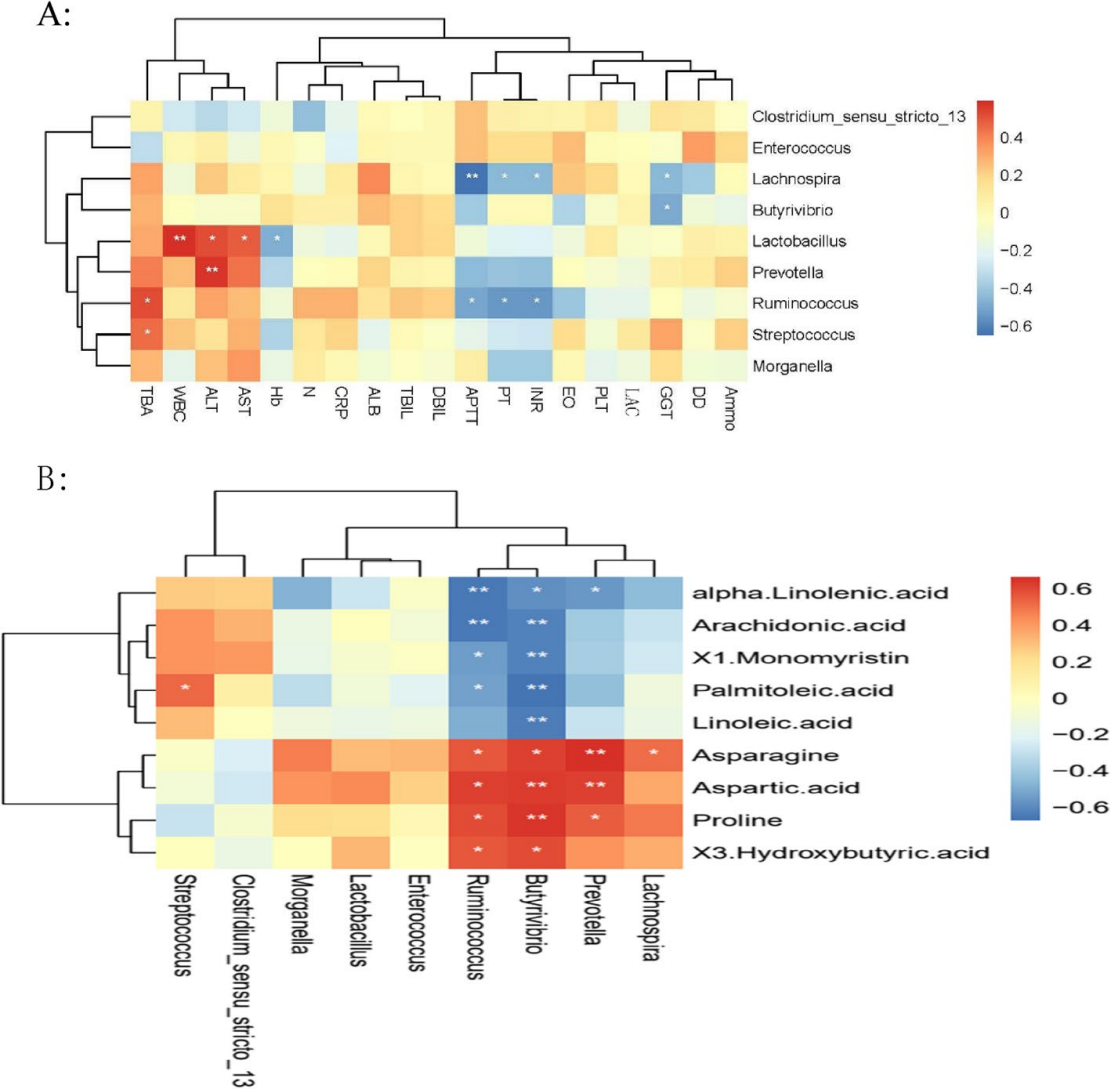
Correlation of gut microbiota, metabolites, and clinical indicators in healthy infants (CON) and infants with infantile cholestasis (IC). Red boxes: positive correlation, blue boxes: negative correlation (*P < 0.05; **P < 0.01). **A** Relationship between differential bacterial genera and differential metabolites in the two groups; **B** Relationship between differential bacterial genera and clinical indicators in the two groups

To better understand the significance of gut microbiota and metabolites in IC, we first identified the correlation between the main differential bacterial taxa and metabolites in the intestine between the IC and CON groups. The results showed that the abundance of *Eubacterium*, *Intestinibacter*, *Ruminococcus*, and *Butyrivibrio* were positively correlated with proline, asparagine, and aspartic acid, while it was significantly negatively correlated with the concentration of α-linolenic acid, linoleic acid and arachidonic acid (Fig. 4A).

In addition, we also analyzed the correlation between the gut microbiota of IC group and clinical indicators. The results showed that *Veillonella* and *Streptococcus* were positively correlated with serum bile acid values, *Butyrivibrio* was positively correlated with GGT values, while *Ruminococcus* and *Faecalibaculum* were negatively correlated with APTT, PT and INR (*P* < 0.05) (Fig. 4B**)**. According to the correlation analysis of metabolites and clinical indicators, we found that *Ruminococcus* and *Butyrivibrio* may play an important role in infant cholestatic liver disease.

## Discussion

IC is attributed to increased concentrations of bile substances (bilirubin, bile acid, and cholesterol) in the liver, blood, and extrahepatic tissues, resulting from hepatocellular injury during bile formation or obstruction of intrahepatic or extrahepatic bile ducts [12]. Gut microbiota play important roles in the onset and development of IC by regulating bile acid metabolism and immune response [13]. As the microbe-rich intestine represents a major hepatic blood supply and an intrinsically close association has been described between gut microbiota and the liver, the modulation of gut microbiota represents a potential therapeutic target against IC [14].

Pediatric patients with cholestasis have a lower gut microbiota diversity, greater fluctuations in gut microbiota composition, and significantly higher bacterial richness than healthy controls [6, 15]. This reduced diversity may be associated with bile acids and gut microbiota dysbiosis [1, 16, 17]. However, our analysis of gut microbial distribution in infants via HTS of 16S rRNA showed no significant differences in the species richness or diversity of gut microbiota between the IC and CON groups, which was inconsistent with existing research [6, 15]. This may be related to the small sample size of our study group, the limited age range (within 6 months), or the exclusivity of breastfed and vaginally-born infants. Indeed, feeding and delivery methods reportedly impact the gut microbiome composition of infants [18, 19]. However, previous cross-sectional studies did not implement screening restrictions related to delivery or feeding modes [2, 6, 15]. Although there is no significant difference in diversity between the two groups in this study, the abundance of certain bacterial genera differs between the two groups.

The results of this study showed that at the genus level, the IC group infants had significantly reduced abundances of *Ruminococcus*, *Butyrivibrio*, *Eubacterium coprostanogenes group*, *Faecalibaculu*n, *Lachnospiraceae*. Previous studies have shown that *Ruminococcus* and *Butyrivibrio* are involved in the oxidation of bile acid hydroxyl and the production of Ursodeoxycholic acid, while the *Eubacterium coprostanogenes group* contains bile salt hydrolase (BSH), which affects the metabolism of bile acid [20–22]. This is consistent with the discovery in this study that the abundance of bacteria involved in bile acid metabolism in the intestine of children with cholestatic liver disease is reduced. The pathway enrichment analysis of microbiota based on 16S rRNA sequencing showed that apoptosis pathways were highly expressed in the seven patients with IC with elevated concentrations of transaminases (more than twice that of the normal range; *P* < 0.05). Consistent with our findings, other studies have demonstrated that the hepatic accumulation of hydrophobic bile salts during cholestasis induces hepatocyte and cholangiocyte apoptosis; hepatocyte apoptosis is accompanied by elevated serum concentrations of transaminases. Moreover, apoptosis is involved in the “death,” not necrosis, of hepatocytes [23, 24]. Therefore, the discovery of specific bacterial genera that trigger anti-apoptotic pathways may be conducive to preventing hepatocyte death.

Patients with IC of unknown etiology also exhibited significantly increased concentrations of intestinal fatty acids, primarily including long-chain fatty acids, i.e., α-linolenic, linoleic, and arachidonic acids, compared with healthy controls. Despite being sources of essential fatty acids and calories, long-chain fatty acids are metabolized in the liver before being absorbed and utilized by the human body. Therefore, when hepatocellular injury occurs in IC infants, fatty acid metabolism and absorption are impacted, which will affect protein and carbohydrate metabolism [25]. This may account for the malnutrition symptoms frequently observed in infants with IC. The correlation analysis of differential bacterial genera and metabolites further revealed that *Ruminococcus* and *Butyrivibrio* negatively correlated with α-linolenic acid (*P* < 0.05), studies have shown that the change of α-linolenic acid content in the intestines may be related to the abundance of *Ruminococcus *[26, 27]. Hence, we speculate that an increase in the abundance of *Ruminococcus* exhibits a positive regulatory effect on improving intestinal homeostasis, reducing the hepatic accumulation of α-linolenic acid pathway metabolites, and improving metabolic pathways of non-essential fatty acids, but the specific mechanism is not yet clear. Moreover, the differential metabolites in patients with IC were found to be enriched in α-linolenic acid metabolites and linoleic acid metabolites (*P* < 0.01). The metabolites of linolenic acid, i.e., eicosapentaenoic acid (EPA) and docosahexaenoic acid (DHA) alleviate the symptoms of non-alcoholic fatty liver disease in children by increasing the activity of ALT and AST, while reducing the inflammatory response in hepatocytes and liver fat deposition [28]. However, the impaired hepatocyte function in patients with IC affects the metabolism of linolenic acid and DHA synthesis in the liver, leading to metabolite accumulation, which may aggravate abnormal inflammatory responses in hepatocytes. Therefore, early enhancement of enteral nutrition and amino acid intake, as well as reduced intake of long-chain fatty acids and an increased intake ratio of short- and medium-chain fatty acids is imperative in patients with IC. These measures can improve liver function and nutritional status, while reducing liver burden, and promoting nutrient absorption and the reverse regulation of gut microbiota homeostasis.

A specific correlation was also detected between intestinal bacteria and significant changes in the intestinal tract of children with IC and their clinical laboratory indexes. More specifically, the serum bile acid value was significantly positively correlated with the content of *Veillonella* in the IC group (*P* < 0.01). Previous studies have shown that *Veillonella* is commonly enriched in AIH, PBC, and PSC [29], and infantile cholestatic jaundice patients showed increased abundances of *Veillonella* [30], which were consistent with the conclusion of our study, and we also found that the abundance of *Veillonella* was negatively correlated with the serum bile acid value in the IC group. Therefore, we speculated that *Veillonella* may be involved in bile acid metabolism or affect its metabolic process, thereby affect the occurrence of liver diseases. *Butyrivibrio* was positively correlated with GGT values. Meanwhile, the coagulation indexes (APTT, PT, and INR) were negatively correlated with *Ruminococcus* abundance in the intestinal tract.

Although the findings of this study provide important insights into the relationship between the gut microbiome and IC, there are certain limitations to this study. Firstly, the sample size is small, as all enrolled children in this study need to meet the requirements of full term, natural delivery, and pure breastfeeding before admission, without a history of antibiotic and probiotic use, or a history of drug use, the purpose is to avoid the influence of other factors such as feeding, but this also leads to a limited number of cases in enrolled children, resulting in a small sample size. Therefore, there may be some impact on the distribution of disease gender, disease severity, and etiology. Additional studies with larger patient groups, are needed to further characterize the relationship between specific bacteria and metabolites.

## Conclusion

In this study, the analysis of gut microbiota and the metabolites from the stool of IC patients revealed that the abundance of some microbiota in the intestinal tract of children with IC differed from that of healthy infants. That is, the abundance of *Streptococcus, Veillonella* and *Clostridium spp.* increased; however, the abundance of *Ruminococcus*, *Butyrivibiro, Eubacterium, Faecaibaculun* and *Lachnospiraceae* decreased significantly. Moreover, the abundance of α- linolenic acid, linoleic acid and arachidonic acid increased in intestinal metabolites in IC patients. *Ruminococcus* and *Butyrivibiro* is associated with changes in metabolites and clinical indexes, *Veillonella* is closely related to bile acid indicators. The changes in gut microbiota composition in children with IC may be related to abnormal inflammatory reactions in their liver. Therefore, this study provides a basis for further analysis of how adjusting gut microbiota composition can promote the treatment of IC children. Of course, it is also necessary to consider the correlation between changes in gut microbiota composition and the already occurring state of bile stasis. Further improvement in clinical trials related to the occurrence and development of bile stasis is needed for verification in the future.

## Materials and methods

### Research subjects

The data for 20 infant patients with IC admitted to the Gastroenterology Department at the Children's Hospital of Capital Institute of Pediatrics, China, from January 2020 to June 2021 were collected. In addition, 20 healthy infants who visited the Department of Health for physical examination from June 2020 to September 2020 were selected as the control patients. This study was reviewed by the Ethics Committee of the hospital (Ethics Review No.: SHERLL2021008), and the guardians of all pediatric participants provided written informed consent.

### Inclusion criteria

The inclusion criteria for the IC group were: (1) infants aged < 1 year; (2) all diagnostic criteria for cholestasis met [4] at admission; (3) no antibiotics or probiotics administered over the preceding 2 weeks; (4) full-term infants born by vaginal delivery; (5) exclusively breastfed before the onset of the disease. The control group comprised infants aged < 1 year with normal health as indicated by physical examinations at the Department of Pediatrics; infants with no other diseases, such as cholestasis or congenital developmental abnormalities; no antibiotics or probiotics administered in the preceding 2 weeks; full-term infants born by vaginal delivery and exclusively breastfed.

### Collection of clinical data

Clinical data for the IC group, including sex, age, and duration of disease, as well as concentrations of transaminases, bilirubin, γ-glutamyltransferase, and bile acid, were collected (first test after admission). Fecal specimens were collected from IC patients within 3 days of admission, those of the control group were collected within 3–5 days of having been confirmed to meet the inclusion criteria. A total of 20 g of each fecal specimen was stored at -80 °C and samples were sent together for testing. Repeated freeze–thaw cycles greatly affect the concentration of microbial-derived metabolites, which are abundant in feces. Therefore, repeated freezing and thawing were avoided throughout the experiments.

### Isolation of DNA

Genomic DNA was extracted from the fecal microbiota. The isolated DNA samples were assessed via electrophoresis on a 1% agarose gel. The concentration and purity of DNA were determined via spectrophotometry. The V3–V4 hypervariable region of the bacterial 16S rDNA gene was amplified with the primer pair 338F (5′-ACTCCTACGGGAGGCAGCAG-3′) and 806R (5′-GGACTACHVGGGTWTCTAAT-3′) via the following amplification steps: initial denaturation (95 °C for 3 min); 27 cycles of denaturation (95 °C for 30 s), annealing (55 °C for 30 s), and extension (72 °C for 45 s); final extension (72 °C for 10 min). The PCR products were separated via gel electrophoresis and recovered using the Axy Prep DNA Gel Extraction Kit (Axygen Biosciences), followed by quantification using the Quantus Fluorometer (Promega, USA). Subsequently, the PCR products were subjected to library construction and sequencing on the Illumina MiSeq platform.

### HTS of 16S rDNA on the MiSeq platform

The raw FASTQ files were first de-multiplexed, then quality-filtered using Chimera_check and merged using FLASH; the sequences were processed using the BIPES protocol.10 and QIIME 1.9. Using the RDP classifier Naive Bayesian classification algorithm to compare and annotate representative sequences with the Silva (V138) database, the annotation information of OUT is obtained. For alpha diversity, the sample data were rarefied, and the Shannon indexes, observed operational taxonomic unit (OTUs) (richness), and Simpson's evenness were calculated. The non-parametric statistical Kruskal–Wallis test was used to assess group differences. To perform beta diversity analysis, we used principal coordinates analysis (PCoA) with weighted Unifrac distances. Permutational multivariate analysis of variance (PERMANOVA) implemented in the adonis function of the R/vegan package (v2.5–2) was then performed to identify group differences based on the weighted UniFrac distances. Differentially abundant taxa were visualized using heatmaps with log2-transformed relative abundances and hierarchically clustered based on Bray–Curtis distance. To further explore key genera that may have contributed to the observed differences in the microbial communities, linear discriminant analysis (LDA) effect size (LEfSe) analysis was performed to estimate the effect size of differentially abundant features with biological consistency and statistical significance. Pathway enrichment analysis was performed using the Kyoto Encyclopedia of Genes and Genomes (KEGG) database by PICRUSt.

### Gas chromatography-mass spectrometry (GC–MS) analysis

After thawing in an ice bath, 50 mg of each sample was transferred into a 1.5-mL centrifuge tube and spiked with 10 µL of the internal standard. After brief mixing, each sample was mixed with 175 μL of pre-chilled methanol/chloroform (v/v = 3/1) for extraction. The mixture was then centrifuged at 14,000 × *g* and 4 °C for 20 min, followed by incubation at -20 °C for 20 min. Subsequently, the supernatant was carefully transferred into an autosampler vial (Agilent Technologies, Foster City, CA, USA) and concentrated for 5 min using a centrifugal vacuum concentrator (Labconco, Kansas City, MO, USA) to remove residual chloroform, followed by lyophilization in a low-temperature freeze dryer (Labconco, Kansas City, MO, USA) prior to gas chromatography-mass spectrometry (GC–MS) analysis. The raw GC–MS data files were subjected to peak extraction, deconvolution, and integration using the XploreMET software (v3.0, Metabo-Profile, Shanghai, China), as well as qualitative analysis using the JiaLab metabolite database. The XploreMET software is currently hosted on Dell Power Edge R730 servers running the Linux Ubuntu 16.10 operating system. The statistical analysis in this study was carried out using a robust R Studio package.

### Statistical analysis

The Kruskal–Wallis rank-sum, Wilcoxon rank-sum, and *t*-tests were employed to assess the significance of differences between the two groups. All statistical analyses in this study were performed using the SPSS software and R packages on Mac OS X. The correlation analysis of clinical indexes was adopted by Spearman's rank correlation coefficient. A *P* < 0.05 was used to indicate statistically significant differences.

### Supplementary Information

**Additional file 1.**

**Additional file 2.**

## Data Availability

The 16S RNA gene sequences are available through the National Genomics Data Center of the China National Center for Bioinformation (CNCB-NGDC) under accession number: Project ID: PRJCA011104; accession number: HRA002824. (https://ngdc.cncb.ac.cn/gsa/browse/CRA009815).

## Abbreviations

IC: Infantile cholestasis
ALT: Alanine amino transferase
AST: Aspartate transferase
GGT: Glutamyl transpeptidase
PT: Prothrombin time
APTT: Activated partial thromboplastin time
INR: International Normalized Ratio
NASPGHAN: North American Society for Pediatric Gastroenterology, Hepatology and Nutrition
TBil: Total bilirubin concentration
DBil: Direct bilirubin concentration
CON: Healthy
BSH: Bile saline hydrolase
EPA: Eicosapentaenoic acid
DHA: Docosahexaenoic acid
