# Supplementary material for: Alterations in gut microbiota and metabolite profiles in patients with infantile cholestasis
Source: BMC Microbiol. 2023 Nov 18;23:357. doi: 10.1186/s12866-023-03115-1 (PMC10656868; doi:10.1186/s12866-023-03115-1)
Supplement: Supplementary file 1 — Additional file 1. [file 12866_2023_3115_MOESM1_ESM.docx]

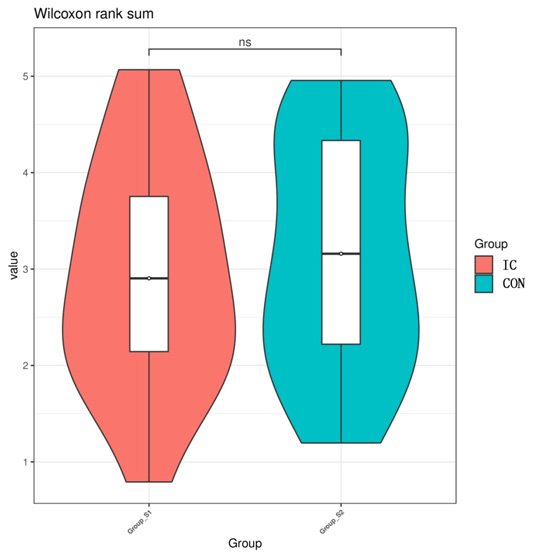


Supplementary Figure 1: The comparison of Shannon and Chao indices of microbial diversity between the IC and CON groups. The red on the left represents the IC group, the blue on the right represents the CON group.
